# Hypericin and Naringenin Exert no Significant Synergistic Apoptotic Effect on Y79 Retinoblastoma Cell Line: Synergistic Effect of Hypericin and Naringenin on Y79

**DOI:** 10.31661/gmj.v13i.3347

**Published:** 2024-04-28

**Authors:** Hamid Zaferani Arani, Zahra Abbasy, Seyed Mahmoud Reza Hashemi Rafsanjani, Nastaran Fooladivanda, Mahdi Kheradmand, Fateme Shariati Far, Dena Saghafi, Amirhossein Shekarriz, Mojdeh Barati, Farzaneh Nasirimotlagh, Fatemeh Ziyadloo, Arefe Nekuifard, Narges Farajee, Mohammadreza Letafat, Marzieh Mehri Tokmeh, Nastaran Teymoorianfard, Bita Massah, Mohammad Amin Javidi

**Affiliations:** ^1^ Department of Surgery, Shiraz University of Medical Sciences, Shiraz, Iran; ^2^ Department of Pediatrics, Tehran University of Medical Sciences, Tehran, Iran; ^3^ Young Researchers and Elite Club, Tehran Medical Sciences, Islamic Azad University, Tehran, Iran; ^4^ Department of Pathology, Shiraz Medical School, Shiraz University of Medical Sciences, Shiraz, Iran; ^5^ Department of Psychology, Tonekabon Branch, Islamic Azad University, Tonekabon, Iran; ^6^ Department of Nursing, Student Research Center, School of Nursing and Midwifery, Isfahan University of Medical Sciences, Internal-surgery Trend, Iran; ^7^ Faculty of Pharmacy, Zanjan University of Medical Sciences, Zanjan, Iran; ^8^ Department of Marine science, Science & Research Branch, Islamic Azad University, Tehran, Iran; ^9^ Faculty of Pharmacy, Tehran University of Medical Sciences, Tehran, Iran; ^10^ Faculty of Medicine, Babol University of Medical Sciences, Babol, Iran; ^11^ Department of Integrative Oncology, Breast Cancer Research Center, Motamed Cancer Institute, ACECR, Tehran, Iran GMJ.

**Keywords:** Retinoblastoma, Herbal Medicine, Apoptosis

## Abstract

**Background:**

According to the anti-cancer impact of hypericin and naringenin, we put the main aim of this study to unravel the apoptotic/anti-cancer effect of these compounds on Y79 retinoblastoma cell line.

**Materials and Methods:**

To calculate the 50%inhibitory concentration (IC50) of hypericin for 24 and 48 hours, XTT assay performed. Cytotoxic effect of naringenin investigated by XTT and trypan blue exclusion assay further confirmed the inhibitory impact of these agents on Y79 cells viability. Flow cytometry Annexin V/PI determined the cell death. The mRNAs expression level of Bax and Bcl-2 investigated by real-time PCR in different groups including the control, cells treated with naringenin, hypericin, or concurrent with both compounds.

**Results:**

The 24 and 48 hours IC50 of hypericin, calculated to be 2.5 and 1.25 (μg/ml), respectively. 50 (μg/ml) naringenin induced about 20% and 30% apoptosis in Y79 cells after 24 and 48 hours. Trypan blue staining and flow cytometry confirmed this data. Moreover, flowcytometry results, revealed that the kind cell death occurred in these cells post treatment was mostly apoptosis. Simultaneous treatment with both agents didn’t show synergistic effect. Bax/Bcl-2 ratio increased in cells treated with hypericin but in cells treated with narigenin didn’t show significant increase in the Bax mRNA level.

**Conclusion:**

Hypericin had more cytotoxic effect in Y79 cells compared with naringenin. Furthermore, hypericin and naringenin didn’t have apoptotic synergistic effect in these cells. According to the real-time PCR results, hypericin induces apoptosis in Y79 cells by disrupt the ratio of Bax/Bcl-2.

## Introduction

Considering the high rate of cancer incidence, chemo preventive agents seem to be much more attractive than before. These are natural or synthetic compounds which hinder tumorigenesis and/or induce apoptosis in cancer cells. Amongst natural chemo preventives we can mention poly phenols, alkaloids, carotenoids, and nitrogen compounds which are reported not to have significant detrimental effect in normal cells. Furthermore, flavonoids are emerging compounds that exist in vegetables, fruits, tea, and etc. and are demonstrated to exert beneficial anti-tumor activities against various cancers [1][2][3][4]. Naringenin is an active biological flavonoid and can be extracted from different citrus including grapefruit. Anti-inflammatory, anti-metastatic, anti-oxidant, and anti-proliferative impacts of naringenin justify its anti-tumor effect especially in breast cancer. Treatment of cancerous cells expressing alpha or beta estrogen receptors, with narigenin induces apoptosis in these cells by activating the mitogen P38 and in a caspase 3 dependent pathway. Further examples of anti-tumor activities of naringenin are available, among them we can mention apoptotic induction in HTP-1 leukemia cell line by disruption in mitochondrial membrane potential, decreasing AKT activity, and triggering caspase cascade; or the same consequence in HepG2 (a delegacy of liver cancer) cell line by up-regulating the ratio of Bax/Bcl2 [5][6][7][8][9][10].

Naringenin treatment of breast cancer cells increased the toxicity of tamoxifen in these cells. Similarly, by up-regulating death receptor in A549 lung cancer cell line, naringenin increased apoptotic effect of tumor necrosis factor in them. Frydoonfar et al revealed that naringenin significantly encumbers HT29 colorectal cancer cell line’s proliferation. Treatment of B16-F10 melanoma cells with this compound reduced cellular viability and metastasis down to 63% compared with the untreated cells. Moreover, naringenin obstructs hepatocarcinoma from N-nitrosodiethylamine (NDEA) and hinder metastasis in cancerous rats’ models. Recently, Lou and colleagues revealed that by hindering Vimentin, N-candherin, MMP-2, and MMP-9, naringenin inhibits invasion and/or metastasis of pancreatic cancer cells [11][12][13][14][15][16][17]. Hypericum perforatum L. also renowned as Tipton Amber, Hardhay weed, Klamath weed, Goat weed, and St.John’s wort is a precious herbal remedy from Clusiaceae or Hypericaceae family, and is aborigine of European and Eastern countries. Various biological compounds can be found in this plant including hyperforin and hypericin. Mounting studies unravel medicinal benefits of hypericin including its anti-bacterial, anti-inflammatory, anti-depression, anti-cancer effect on solid tumors and hematologic malignancies, and etc. [18][19][20]. Hypericin induces apoptosis in caspase dependent manner, activates caspases 3 and 9 in MT450 carcinoma cells; this is confirmed, when cells treated by zVAD.fmk, a caspase inhibitor, the apoptosis induction obstructed [21][22][23][24][25].

Retinoblastoma is an eye cancer which is more common in children and rarely occurs in adults. It begins from retina of eye which is composed of different cells including nerve cells that sensing light and transmit signals to brain where they are interpreted. Although it is rare there is no decisive cure for this cancer and when happens, irreparable consequence of losing 1 or both eyes follows [26][27][28][29][30]. In this study our main aim was to investigate the inhibitory/apoptotic effect of hypericin and naringenin on the Y79 retinoblastoma cells, we further examined whether these 2 known remedies exert synergistic impact on Y79 when used simultaneously.

## Materials and Methods

### Cell Culture

Y79 cell line purchased from Pasteur Institute of Iran. These cells were cultured in RPMI medium supplemented with 10% fetal bovine serum (FBS) and 1% antibiotics (penicillin+streptomycin) (all from Gibco, United states). Cultures cells were incubated in 37 °C, 5% CO2, and 95% air. The medium of cells were replaced with fresh medium twice a week.

### XTT Assay

To investigate the anti-proliferative/ death induction of hypericin and naringenin effect on Y79 cells, XTT assay performed. For this aim 10000 cells were transferred into each well of 96-well plate; 24 hours later, these cells were treated with concentration range (2-100 μg/ml) of hypericin or naringenin (both from Sigma, Germany) for 24 and 48 hours (each concentration was at least in triplicate). After that, XTT (Sigma, Germany) solution added to each well according to the manufacturer’s instruction. Finally, the absorption of each well calculated at 570 nm by ELISA reader (BioTek, United states).

### Flowcytometry

Determination of cellular death type occurred after hypericin and/or naringenin treatment, Annexin V/ Propidium Iodide (AV/PI) (Roche, Switzerland) flow cytometry performed. For this aim, after treatment period, Y79 cells centrifuged and the above medium discarded; solutions of the AV/PI kit added to these cells exactly the same way of the manual’s instruction of the kit and analyzed with BD FACS Calibur (BD biosciences) device. Population of cells with low AV/low PI reported as live cells, that of with high PI/ low AV reported as necrotic, with high AV/low PI as early apoptotic, and with both reporters high considered as late apoptotic.

### Trypan Blue Exclusion

To further confirm cellular death in treated cells, trypan blue staining and counting of cells performed. Viable cells membranes do not let the dye to pass through and hence dead cells become blue and viable cells retain unstained. To this end, 10μl of cellular suspension mixed with 10μl of 4% trypan blue dye (Sigma, Germany). These cells were then counted by the aid of neobar lam and invert microscope (Olympus, Japan). The percentage of viable cells was determined with the following formula: (number of viable cells/numbers of whole cells) *100.

### RNA Extraction and cDNA Synthesis

Total RNA extracted utilizing TRizol reagent (Invitrogen, India). Concentration and plausible contamination of the extracted RNAs were examined by a nanodrop device (Thermo scientific, United states). 1μg of total RNA used as template for cDNA synthesis (Vivantis, Malaysia). These cDNAs were as template for the next step, real-time PCR.

### Real-time PCR

We further tried to investigate the impact of hypercin and/or naringenin treatment on the Y79 cells at molecular level. To this end expression level of Bax (pro-apoptotic gene) and Bcl2 (anti-apoptotic gene) at mRNA level in different samples examined by real-time PCR. Primers designed to specifically amplify cDNA of the mentioned genes (βactin utilized as internal control) (primers’ sequence are mentioned in Table-1). Real-time PCR reactions performed at triplicate with SYBR green master mix (Ampliqon, Denmark) in ABI 7500 real-time PCR device. The program was as follow: Holding Stage: 95 °C/15 minutes; Cycling Stage: denaturing step: 95°C/15 s, followed by annealing step 60 °C/30 s, amplification step 72°C/30s (Number of Cycles: 40). 3- Melt curve analysis stage. To evaluate the relative expression of the mentioned genes, 2 -ΔCt used. Furthermore, the fold change calculated with the formula 2 -ΔΔCt

### Statistical Analysis

All statistical analysis performed by Graphpad Prism V.8. In cases we intended to compare results of two groups, unpaired student t-test done, and p values less than 0.05 considered as significant.

## Results

**Figure-1:**
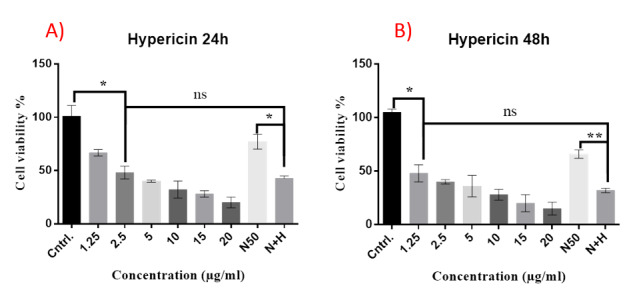
Figure 1. XTT assay. XTT results revealed that the 24 (A) and 48 (B) hours IC50 of hypericin in Y79 cells are 2.5 and 1.25 (μg/ml) respectively. Simultaneous treatment of these cells with 50 (μg/ml) of naringenin and IC50 of hypericin didn’t decrease cell viability significantly compared with the hypericin IC50.

**Table:** Table1. Primers’ Sequence Utilized for Real-time PCR

Gene	Forward primer	Reverse primer	Amplicon length
GAPDH	AAGTTCAACGGCACAGTCAAGG	CATACTCAGCACCAGCATCACC	121 bp
Bax	CCAAGAAGCTGAGCGAGTGT	CCCAGTTGAAGTTGCCGTCT	156 bp
Bcl-2	TCTTTGAGTTCGGTGGGGTC	GTTCCACAAAGGCATCCCAG	153 bp

### Calculating the IC50

Treatment of Y79 cells with concentration range of hypericin for 24 and 48 hours, unraveled the 50 % inhibitory concentration (IC50). According to the XTT data, the 24 hours and 48 hours IC50 of hypericin in these cells calculated to be 2.5 and 1.25 (μg/ml) respectively. Treatment of Y79 cells with 50 (μg/ml) of narigenin induced about 75 and 65 % cell death after 24 and 48 hours, respectively. Simultaneous treatment of cells with the corresponding IC50 of hypericin and 50 (μg/ml) of narigenin for 24 and 48 hours didn’t show significant synergism on cell death, and cell viability didn’t decrease statistically significantly compared with cell viability of cells when treated with IC50 of hypericin (Figure-1).

In the following experiments, in cases not mentioned, the 24 hours IC50 dose of hypericin utilized (named as H sample); or with 50 (μg/ml) of narigenin for 24 hours (N samples); or simultaneously with both (HN sample).

### Cell Count Change

Treatment of Y79 cells with the IC50 of hypericin calculated from the data obtained from the XTT assay, affected cell count under light microscope and seemed to inhibit proliferation of these cells.

**Figure-2:**
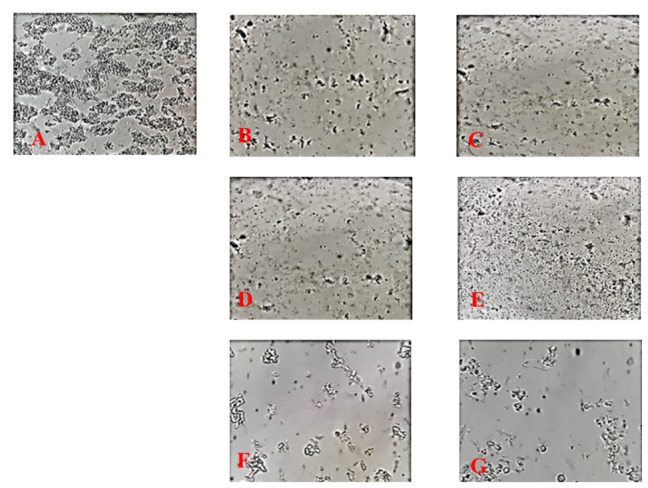
Figure 2. Decreasing in the cell count of Y79 cells after different treatments. A) control, untreated Y79 cells; B) treated with 2.5 (μg/ml) of hypericin for 24 hours; C) cells treated with 1.25 (μg/ml) of hypericin for 48 hours; D) treated with 2.5 (μg/ml) of hypericin and 50 (μg/ml) of nairingenin for 24 hours; E) treated with 1.25 (μg/ml) of hypericin and 50 (μg/ml) narigenin for 48 hours; F) treated with 50 (μg/ml) of naringenin for 24 hours; and G) treated with 50 (μg/ml) of naringenin for 48 hours. (Magnification 200X.)

Cells death was obvious in the simultaneous treatments (24 and 48 IC50 of hypericin and 50 (μg/ml) of naringenin). Cellular death was much less under microscope in the samples treated with and 50 (μg/ml) of naringenin for 24 and 48 hours (Figure-2).

### Investigating Pro/Anti-apoptotic Genes Expression

**Figure-3:**
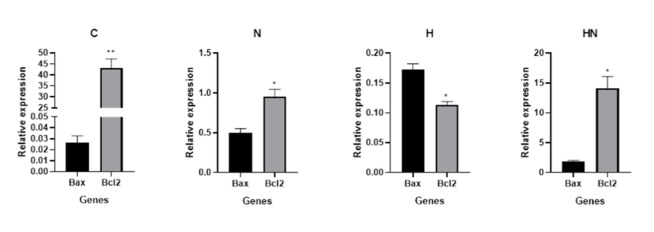
Figure 3. Real-time PCR results. Data obtained from real-time PCR, demonstrated that the expression of Bcl-2 gene at mRNA level was much higher than the Bax gene in the control/untreated sample (C). After treatment of Y79 cells with naringenin Bcl-2 mRNAs expression level was still higher than the Bax gene (N); however, hypericin treated Y79 cells expressed Bcl-2 mRNA less than Bax (H). The expression level of Bcl-2 mRNA was more than the Bax in samples treated with hypericin and naringenin simultaneously (HN).

**Figure-4:**
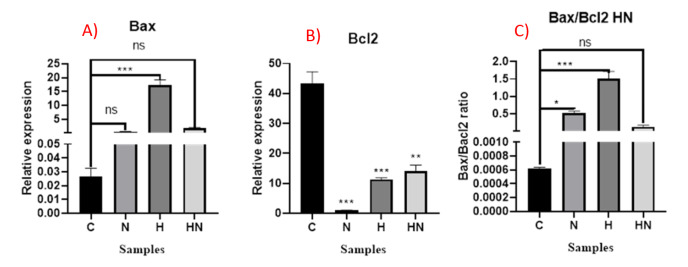
Figure 4. Comparing Bax, Bcl-2 expression, and Bax/Bcl-2 ratio in different samples. The Bax mRNA level increased significantly after hypericin treatment (A); Bcl-2 mRNA level decreased (B) after treatment of Y79 cells with hypericin, naringenin, and simultaneous treatment with naringenin and hypricin. The Bax/Bcl-2 ratio increased after treatment of the Y79 cells with naringenin or hypericin but not after treatment of these cells with both of the agents simultaneously (C).

Investigating the expression of pro-apoptotic Bax or anti-apoptotic Bcl-2 mRNAs revealed alteration of these genes expression in treated samples. After treatment of Y79 cells with hypericin, naringenin, or simultaneously, Bax expression level increased, and Bcl-2 expression decreased compared in all treated samples compared with the Y79 control/untreated cells (however this increasing was not statistically significant in the cells treated with naringenin or simultaneously with both reagents). The Bax/Bcl-2 ratio modified significantly in samples treated with hypericin and not in the other samples (Figures-3 and -4).

### Studying Apoptosis Induction

**Figure-5:**
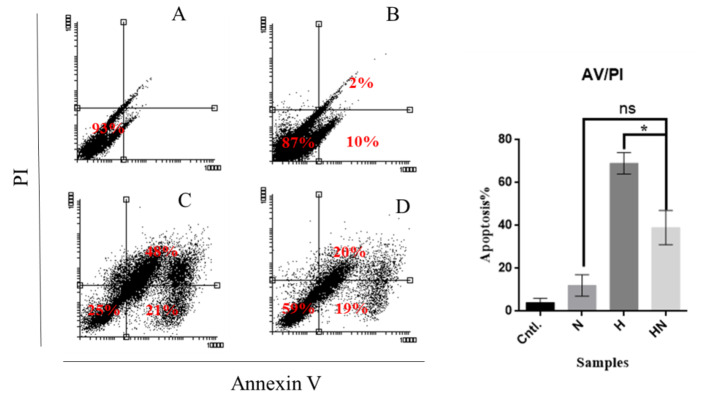
Figure 5. Flow cytometry results. Annexin V/ PI flow cytometry data demonstrated that treatment of Y79 cells with naringenin induced about 12% apoptosis (B); cells treated with hypericin induced about 65% apoptosis in these cells (C); cells treated with hypericin and naringenin simultaneously revealed approximately 40 % apoptosis (D). A) Control/untreated Y79 cells, left bottom quadrant: live cells, right bottom: early apoptosis, left top: necrosis, and right top quadrant: late apoptosis.

AV/PI flow cytometry results revealed that hypericin or naringenin induces apoptosis in treated Y79 cells. Samples treated with hypericin underwent much more apoptosis compared with other samples (Figure-5).

## Discussion

Naringenin and hypericin both are herbal remedies which have been so attractive to the researchers especially in field of cancer. Their anti-cancer properties, at least in some parts, may results from the anti-oxidant characteristics of both agents [31]. Based on the present study, hypericin and naringenin affect Y79 retinoblastoma cells’ survival in a dose dependent. This phenomenon reported in other cancer types but not in Y79 cells. Based on the XTT assay of the current study, 2.5 and 1.25 (μg/ml) of hypericin induces about 50% cell death and according to the AV/PI flow cytometry results, the kind of death was mostly apoptosis. On the other hand, naringenin with the dose of 50 (μg/ml) induces about 12% apoptosis; which may declare that the cytotoxicity of hypericin is much higher in Y79 cells compared with naringenin. Trypan blue staining experiment confirmed these results. In a recent published study, Tondro et al. assessed the possible role of mesenchymal stem cells derived conditioned media, as a delegacy of their secretome, on the proliferation and/or drug resistance of a tongue cancer cell line. They showed that these conditioned media can induce both properties in the mentioned cancerous cells. Furthermore, they evaluated whether hypericin can overcome this resistance or not. They showed that hypericin with the concentration of 20 µg/ml after 48 hours can effectively down-regulate the expression level of ABCB1 and ABCG2, genes that involves in drug resistance [ref: Inhibition by Hypericin of Tongue Squamous Carcinoma Cell Proliferation and Treatment of Resistance in Dental Pulp Stem Cells]. Although they found that hypericin has cytotoxicity and anti-cancer effect in tongue cancer, the calculated IC50 for their study was more than that obtained in our results; this reflects the different response of cancerous cells to the hypericin treatment. This phenomenon at least in some steps may be due to the different genetic pattern of the treated cells; Abbasi et al. revealed that the P53 status may be a key factor in this response. They showed that breast cancer cells with wild type P53 are more sensitive in treatment with hypericin [ref: Hypericin Induces Apoptosis in MDA-MB-175-VII Cells in Lower Dose Compared to MDA-MB-231].

In a study performed by Tatto and colleagues, they revealed that naringenin induces apoptosis in a caspase 3 dependent manner by activating P38 mitogen [10]. Although these apoptosis pathways are interrelated, but hypericin seems to induce apoptosis in a somehow different pathway compared with naringenin. By this view of point we may interpret why simultaneous treatment hypericin and naringenin didn’t exert synergistic anti-cancer effect on Y79 cells. Flow cytometry AV/PI results revealed that simultaneous treatment of the Y79 cells with hypericin and naringenin induced significantly less apoptosis compared with the samples treated with just hypericin. This in some aspects unravels that the molecular pathways by which these two agents induce apoptosis, may not be so aligned. Ghiasvand et al. showed the anti-neoplastic effect of hypericin on a glioblastoma cell line. The IC50 of hypericin on U87 gliobastoma cell line revealed to be 1.5 µg/mL which is in concurrent with the results obtained here. By using next generation sequencing, they found 312 DEGs after treatment of U87 cells with hypericin; the upstream modulators of these DEGs were related with GBM stem cell transcription factor [ref: Transcriptome analysis evinces anti-neoplastic mechanisms of hypericin: A study on U87 glioblastoma cell line].

Real-time PCR data further confirmed this data, according to our study, naringenin induces apoptosis in Y79 cells independent of Bax and Bcl-2 pathway; on the other hand, hypericin are dependent on the Bax and Bcl-2. Jin CY et al demonstrated that ectopic expression of Bcl-2 affects naringenin-dose dependent apoptosis induction and resembles when using caspase3 or 9 inhibitors. Their experiments further declare that, over-expression of Bcl-2 attenuates naringenin induced Bax translocation and cytochrome C release to cytosol in human leukemia U937 cells [14]. Our results, however is not aligned with this data, after treatment with hypericin, Bax/Bcl-2 ratio modified significantly compared with the control sample; this increasing, can violate the 1 to 1 ratio of Bax to Bcl-2. This can derive the Bax-Bax dimers formation instead of Bax-Bcl-2 and infer apoptosis by the aid of mitochondria. This discrepancy may in some parts arise from different cell lines utilized for experiments. This finding may be in concurrent with the study performed by Piryaei et al. in which they assessed the underlying molecular mechanism of action of anti-cancer effects of hypericin in B-CPAP and TCP-1 cells. They demonstrated that hypericin can induce apoptosis in these cells in an extrinsic caspase dependent pathway. They further investigated the possible anti-metastatic effect of hypericin on the mentioned cells. They revealed that the expression level of genes especially CDH1 and LGALS3 altered after hypericin treatment.

## Conclusion

In conclusion, hypericin and naringenin, according to our experiments, induces apoptosis in Y79 retinoblastoma cells, and the toxicity of hypericin is much more. Although more complementary experiments including in vivo/ex vivo studies are required; we didn’t see synergistic anti-cancer effect of these two agents in Y79 cells. We should mention that this study suffers from some limitations including lack of in vivo experiments to confirm the obtained data.

## Conflict of Interest

Authors declare they don’t have competing interests.
